# MULTILINGUAL THEMATIC ANALYSIS USING VARIED TRANSLATIONS AND LINGUISTIC VALIDATION OF CODES: ADVANCING INCLUSION

**DOI:** 10.1093/geroni/igac059.610

**Published:** 2022-12-20

**Authors:** Orly Tonkikh, Elena O Siegel, Anna Zisberg, Nurit Gur-Yaish, Ksenya Shulyaev, Amos Rogozinski, Heather Young

**Affiliations:** University of California Davis, Sacramento, California, United States; University of California Davis, Sacramento, California, United States; University of Haifa, Israel, Haifa, Hefa, Israel; Oranim Academic College of Education, Tivon, Hefa, Israel; University of Haifa, Haifa, Hefa, Israel; University of Haifa, Haifa, Hefa, Israel; University of California Davis, Sacramento, California, United States

## Abstract

Despite the widely acknowledged cultural diversity among older adults and family caregivers, the representation of people from different linguistic backgrounds in a single study is rare. Single language is a common inclusion criterion, limiting the diversity of samples. Performing cross-cultural qualitative research using several languages within a study requires a systematic data harmonization approach to assure the trustworthiness of the analysis. This paper describes strategies used to establish trustworthiness in the multi-lingual thematic analysis of a dyadic qualitative descriptive study of older adults hospitalized in general hospitals and family members who accompanied them during the hospitalization. Participant interviews (n=22) were conducted in English, Hebrew, and Russian according to individual preferences. Four of the dyads (8 participants) were interviewed in different languages. Based on the template analysis approach, we performed multiple multi-lingual translations and linguistic validation of an inductively identified high-level coding scheme. Each linguistic validation process included a reconciliation of two forward translations and harmonization using back translation, performed for each pair of languages. We describe the rationale for decisions regarding the translation process (the timing of translation after establishing a high-level coding scheme, using a hermeneutic translation approach to achieve conceptual equivalence, variability in the socio-demographic context of the translators, recruitment of both translators and linguists). This study provides principles of a feasible systematic approach to overcome linguistic barriers in caregiving research, providing an avenue for inclusive research among multi-cultural and multi-lingual study samples.

